# Comparison of the intestinal microbiota composition and function in healthy and diseased Yunlong Grouper

**DOI:** 10.1186/s13568-019-0913-3

**Published:** 2019-11-21

**Authors:** Chao Ma, Chunxiu Chen, Lei Jia, Xiaoxu He, Bo Zhang

**Affiliations:** Tianjin Bohai Sea Fisheries Research Institute, Tianjin, People’s Republic of China

**Keywords:** 16S rRNA, Intestinal microbiota, PICRUSt, Yunlong Grouper

## Abstract

Maintaining stabilization of the intestinal microbiota is important in preventing bacterial diseases in cultured fish. At present, there have been no reports on the composition and functional analysis of intestinal microbiota in Yunlong Grouper (*Epinephelus moara♀ *× *Epinephelus lanceolatus♂*). In this study we analyzed and compared the intestinal microbiota composition of healthy and diseased pond-reared fish to discern the functional profile of a healthy status. The richness and diversity of the intestinal microbiota did not differ significantly between diseased and healthy fish, yet the abundance of predominant phyla like the *Proteobacteria* were upregulated in the diseased Yunlong Grouper. At the genus level, a significant reduction of *Cetobacterium* was observed in the intestinal tracts of diseased fish, as *Pseudomonas* became the most dominant bacterium. To compare the intestinal microorganism abundances between the two health groups of fish, we first screened the gut bacteria and discerned 4 phyla and 12 genera to designate a healthy status in Yunlong Grouper. The environmental bacterial community influenced composition of the intestinal microbiota in Yunlong Grouper, and the intestinal microbiota of diseased fish was more susceptible to the influence of the culture water. In addition, the prediction of functional genes by phylogenetic investigation of communities by reconstruction of unobserved states (PICRUSt) indicated that the intestinal microbiota of Yunlong Grouper is related mainly to the terms “metabolism, environmental information processing, genetic information processing, human diseases, and cellular processing; moreover, the functions of the intestinal microbiota differed between the different health states of this fish. The overall results indicate that the occurrence of disease can affect the composition and function of the intestinal microbiota in a cultured fish.

## Introduction

The intestinal microbiota comprise a complex and diverse ecosystem attached to the intestinal mucosa and they exist in a dynamic state. Once the balance of the intestinal microbiota is destroyed, the host becomes susceptible to innumerable bacterial diseases. Thus, stability of the intestinal microbiota community is an important factor in preventing bacterial diseases in animals (Round and Mazmanian 2009; Ringø et al. 2003). Likewise in fishes, the intestinal microbiota play an important role in growth and development, nutrient absorption, immunity and disease resistance (Eddy and Jones 2002; Wu et al. 2013. However, unlike in terrestrial animals, the fish gut is directly coupled to its aquatic environment. The formation of intestinal microflora in cultured fish is not only related to the species of fish, but is also affected by the aquatic environment, food, and other factors(Pérez et al. 2010). Therefore, knowledge of the intestinal and environmental microbiota has great significance for preventing disease and achieving healthy growth in cultured species.

Yunlong Grouper is a hybrid progeny (*Epinephelus moara♀* × *E. lanceolatus♂*) with fast growth, a high survival rate, and wide temperature range. Researchers have reported on their embryonic (Tian et al. 2017a) and post-embryonic development (Wu et al. 2016a, b, c), the adaptability of fry to temperature and salinity (Xing et al. 2017), and karyotype analysis (Cheng et al. 2018). However, there has been no research using 16S rRNA gene sequencing to analyze the intestinal microflora of Yunlong Grouper in different health states, nor studies of the correlation between composition of the intestinal microflora and the culture environment.

Phylogenetic investigation of communities by reconstruction of unobserved states (PICRUSt) is a bioinformatics software package used to predict metagenomic functional genes (Morgan et al. 2013). It can predict the metabolic function of bacteria and archaea through 16S rRNA gene sequences, and its applications are growing. For example, in one study (Wu et al. 2016a, b, c), PICRUSt predicted the oral flora of smokers and non-smokers, and found that there were significant differences in 83 functional metabolic pathways. Loudon (Loudon et al. 2014) used PICRUSt to identify core bacterial communities on the skin of red-backed salamanders. Functional predictions have revealed that a core bacterial community is closely linked to immune regulation and plays an important role in maintaining the basic functions of the body.

At present, the potential relationship between changes in the composition and the function of the intestinal microflora in this species remain unclear. Therefore, this study used healthy and diseased Yunlong Grouper and samples of their culture water and feed as research objects. 16S rRNA sequencing technology was used to analyze the composition of the intestinal microbiota in different health states of the fish. PICRUSt was applied to predict gene function, evaluate changes in the intestinal microbiota function, and study the correlation with the culture environment. Furthermore, from the perspective of micro-ecology, this investigation of the influence of disease on the composition and function of the intestinal microbiota of Yunlong Grouper will provide a scientific basis and reference for disease prevention and the healthy breeding of Yunlong Grouper.

## Materials and methods

### Sample collection

Yunlong Grouper and their feed and culture-water were sampled at the Tianjin Xingsheng Sea and Freshwater Aquaculture Co., Ltd. All sampled fish were of the same age (6 months old) and had been fed the same feed and reared in a similar environment (n = 3 per group). Two experimental groups were acquired from same recirculating aquaculture systems: healthy fish (H group) and diseased fish (D group); the latter were taken from a culture pond specifically maintained for producing diseased fish, and all the fish had obvious signs of disease by visual observation. The healthy samples were randomly removed from a culture pond and visually observed for signs of disease before being dissected. Mean (± SD) of body length (BL) was 38.33 ± 3.79 cm for the H group vs. 30.67 ± 1.53 cm for the D group (*t* test, *P *= 0.12); body weight (BW) was 684.13 ± 124.24 g for the H group vs. 374.23 ± 71.02 g for the D group (*t*-test, *P* = 0.11). There were three replicates for each of the two health-status groups. Sterile water-collection bottles (2 L) were used to take culture water from four locations in each of the two 20-m^3^ culture ponds. Feed samples were collected using sterile bags. All samples were immediately transported to the laboratory.

### DNA extraction and sequencing of 16S rRNA genes

Prior to autopsy, the skin of sampled fish was disinfected with 70% ethanol. The gut was removed and placed into a 1.5-mL centrifuge tube, and immediately stored at − 80 °C until DNA extraction. DNA was extracted from the intestinal contents using a QIAamp DNA Stool Mini Kit (Qiagen, Germany), according to the manufacturer’s instructions. The 2-L water samples were sequentially filtered through 0.22-μm filter paper (Jin Teng, China) to collect as many bacterioplankton as possible; DNA was extracted from these organisms using the E.Z.N.A.^®^ Water DNA Kit (Omega Bio-Tek, Norcross, GA, USA) according to the manufacturer’s instructions. The V4–V5 variable region of 16S rRNA genes of gut microbiota were amplified by polymerase chain reaction (PCR) using the primers 515F [5′-GTGCCAGCMGCCGCGGTAA-3′ (Caporaso et al. 2011)] and 926R [5′-CCGTCAATTYYTTTRAGTTT-3′ (Liu et al. 2007)]. Next, the total bacterial DNA of each sample, extracted by the above method, was constructed by a two-step PCR amplification method. The first PCR reaction reaction conditions were 94 °C for 2 min; and 94 °C for 30 s, 56 °C for 30 s, 72 °C for 30 s, 72 °C for 5 min, 25 cycles. The second PCR conditions were 94 °C for 2 min; and 94 °C for 30 s, 56 °C for 30 s, 72 °C for 30 s, 72 °C for 5 min, 10 °C heat preservation, for 8 cycles.

All PCR products were recovered from the gel with an AxyPrep DNA Gel Recovery Kit (Axygen, USA) and then quantified using an FTC-3000™ Real-Time PCR system. The amplicons were sequenced with Illumina MiSeq 2 × 300 bp (Illumina Inc., San Diego, CA, USA). Sequencing was carried out at TinyGene Bio-Tech Co., Ltd (Shanghai, China).

### Statistical methods

All sequences were clustered into operational taxonomic units (OTUs) by de novo OTU picking at the 97% similarity level, performed with the software Mothur (Schloss et al. 2009). The Ribosomal Database Project (RDP) Classifier (Version 2.2; https://sourceforge.net/projects/rdp-classifier) and the Greengenes Database (http://greengenes.lbl.gov) were used for taxonomic assignments. Rarefaction curves and Alpha diversity indices (i.e. ACE, Chao1, Shannon and Simpson) were generated with Mothur (Schloss et al. 2009). Significant differences of alpha diversity index between groups were calculated with a Kruskal–Wilcox test. Principal coordinates analysis (PCoA) with the weighted UniFrac distance was conducted based on the phylogenetic information (Lozupone et al. 2006). For bar plots and heatmap analysis, the PCoA was calculated and plotted in R (Version 3.4.1), and the hcluster analysis was plotted in QIIME (Version 1.9.1). Statistical differences were analyzed with SPSS 13.0 (SPSS Inc., Chicago, IL, USA) and Metastats (http://metastats.cbcb.umd.edu) software. A *P*-value of less than 0.05 was considered significant.

Characterization of the healthy and diseased Yunlong Grouper in each treatment was performed using linear discriminant analysis (LDA) effect size (LEfSe) method (http://huttenhower.sph.harvard.edu/lefse) for biomarker discovery. The LEfSe method uses the Kruskal–Wallis rank sum test to detect features with significantly different abundances between the assigned taxa and performs the LDA to estimate the effect size of each feature with the normalized relative abundance matrix.

### Functions prediction

PICRUSt was employed to predict the functional profiles of microbial communities (Morgan et al. 2013). After normalized of the 16S rRNA copy numbers, the microbiota functions were predicted with reference to the Kyoto Encyclopedia of Genes and Genomes (KEGG) Orthology (KO) database (Kanehisa et al. 2012).

## Results

### 16s rRNA sequencing data and alpha-diversity analysis

By sequencing the V4–V5 region of the 16s rRNA gene from the samples, the high-throughput sequencing data in each sample can be statistically analyzed. The effective tags of each sample were between 29,297 and 37,177, and the optimized clean data were between 21,091 and 34,280. A total of 521 OTUs were obtained, which identified to 18 phyla. The samples of healthy fish aquaculture water (HW), diseased fish aquaculture water (DW), feed (AF), healthy fish intestinal microbiota (H1, H2, H3), and diseased fish intestinal microbiota (D1, D2, D3), respectively, included 275, 280, 69, 155, 108, 50, 178, 198 and 145 OTUs (Table 1). The number of OTUs were 104.33 ± 52.60 for healthy fish, and 173.67 ± 26.76 for diseased fish; the difference between the two groups was not significant (*P* > 0.05).

**Table 1:** The sequencing depth of each sample

Sample name	Effective tags	Clean data	Numbers of OTUs
HW	35,768	22,173	275
DW	33,639	21,091	280
AF	33,890	25,893	69
H1	29,297	27,344	155
H2	36,262	34,280	108
H3	34,751	32,727	50
D1	37,177	28,683	178
D2	37,025	31,829	198
D3	34,530	27,884	145

**Fig. 1:**
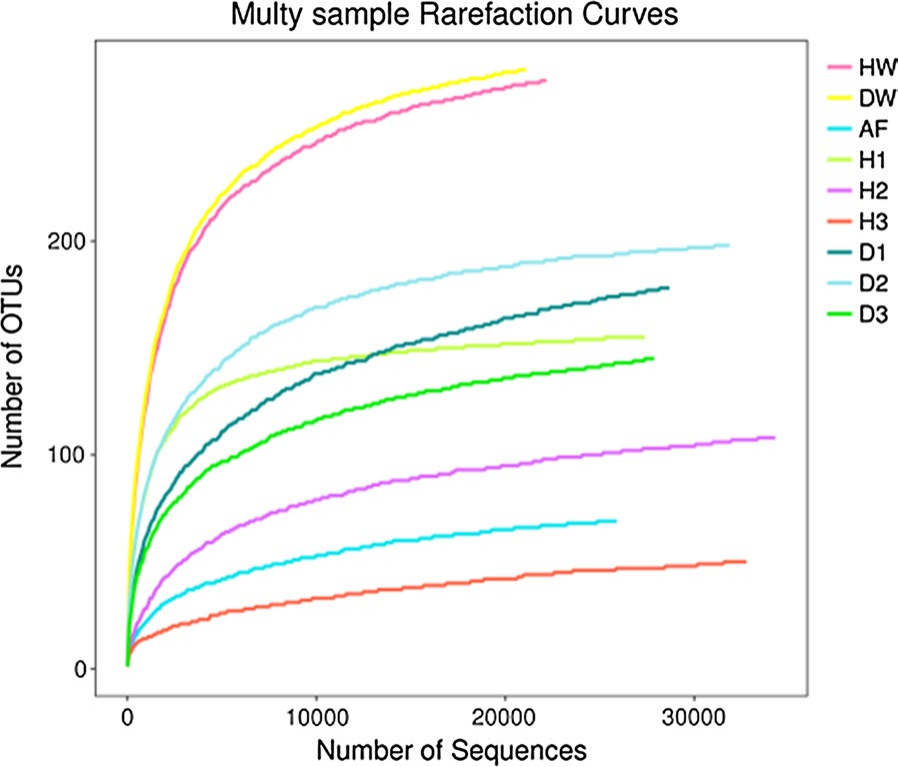
Rarefaction curve. H1–H3 represent the intestinal microbiota of three healthy fish; D1–D3 represent the intestinal microbiota of three diseased fish; *HF* healthy fish aquaculture water, *DW* diseased fish aquaculture water, *AF* feed

The rarefaction curve was drawn with randomly selected sequence numbers and the number of OTUs they can represent (Fig. 1). The results showed that the curve of each sample tended to flatten with higher sequence numbers, indicating that the amount of sequencing data was reasonable. The alpha diversity index of the two groups is shown in Table 2. The Chao1 and ACE indices reflected the species richness of the intestinal microbiota in the samples: these were 118.86 ± 27.74 and 119.53 ± 27.20, respectively, for the healthy fish group, and 194.45 ± 15.75 and 195.69 ± 15.88 for the diseased fish group. The Shannon and Simpson indices reflected the diversity of the intestinal microbiota in the samples: these were 1.80 ± 0.60 and 0.34 ± 0.11, respectively, for the healthy fish group, and 2.56 ± 0.34 and 0.20 ± 0.06 for the diseased fish group. The Wilcox-test was used to analyze differences in the alpha diversity index between the healthy and the diseased fish groups, and the results showed no significance (*P* > 0.05).

**Table 2:** Analysis of alpha diversity indices for the healthy and the diseased groups of fish (mean ± SD)

Index	Healthy group	Diseased group	Wilcox-test P-value
Chao1	118.86 ± 27.74	194.45 ± 15.75	0.081
ACE	119.53 ± 27.20	195.69 ± 15.88	0.081
Shannon	1.80 ± 0.60	2.56 ± 0.34	0.383
Simpson	0.34 ± 0.11	0.20 ± 0.06	0.383

### Composition of the intestinal microbiota

**Fig. 2:**
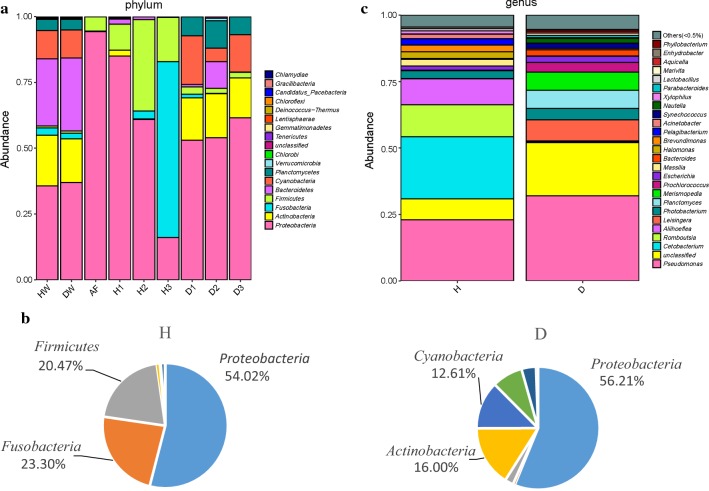
**a** Relative abundance of each sample at the taxonomic level of phylum. **b** Comparison of the relative abundances of the dominant intestinal microbiota phyla between the healthy and diseased Yunlong Grouper. **c** Relative abundance of the dominant genera (mean relative abundance > 0.5%) in the intestinal microbiota between the healthy and the diseased Yunlong Grouper

To estimate the abundance of taxa, the OTUs identified a total of 18 phyla in the fish intestinal microbiota, feed, and culture-water samples. The microbiota composition of the healthy fish aquaculture water was similar to that of the diseased fish aquaculture water. Both showed *Proteobacteria*, *Bacteroidetes*, and *Actinobacteria* as the dominant phyla, and total relative abundance was > 80%. The microbiota composition of the feed was relatively simple, with *Proteobacteria* as the dominant phyla, with a relative abundance of 94% (Fig. 2a). The three most-dominant intestinal microbiota taxa in healthy fish were *Proteobacteria* (54.02%), *Fusobacteria* (23.30%), and *Firmicutes* (20.47%), while that of diseased fish comprised *Proteobacteria* (56.21%), *Actinobacteria* (16.00%), and *Cyanobacteria* (12.61%) (Fig. 2b).

After further analysis of the sample data, each sample of fish intestinal microbiota was screened for the dominant genera (relative abundance > 0.5%), and the microbiota composition and proportions of each group were analyzed at the genus level (Fig. 2c). The most-dominant genera in healthy fish intestinal microbiota were *Cetobacteriu*m and *Pseudomona*s, with a relative abundance of 23%. The dominant genera in the diseased fish group was *Pseudomonas*, with a relative abundance of 32%, whereas the relative abundance of *Cetobacterium* was only 0.5%.

### Sensitive microbial screening

**Fig. 3:**
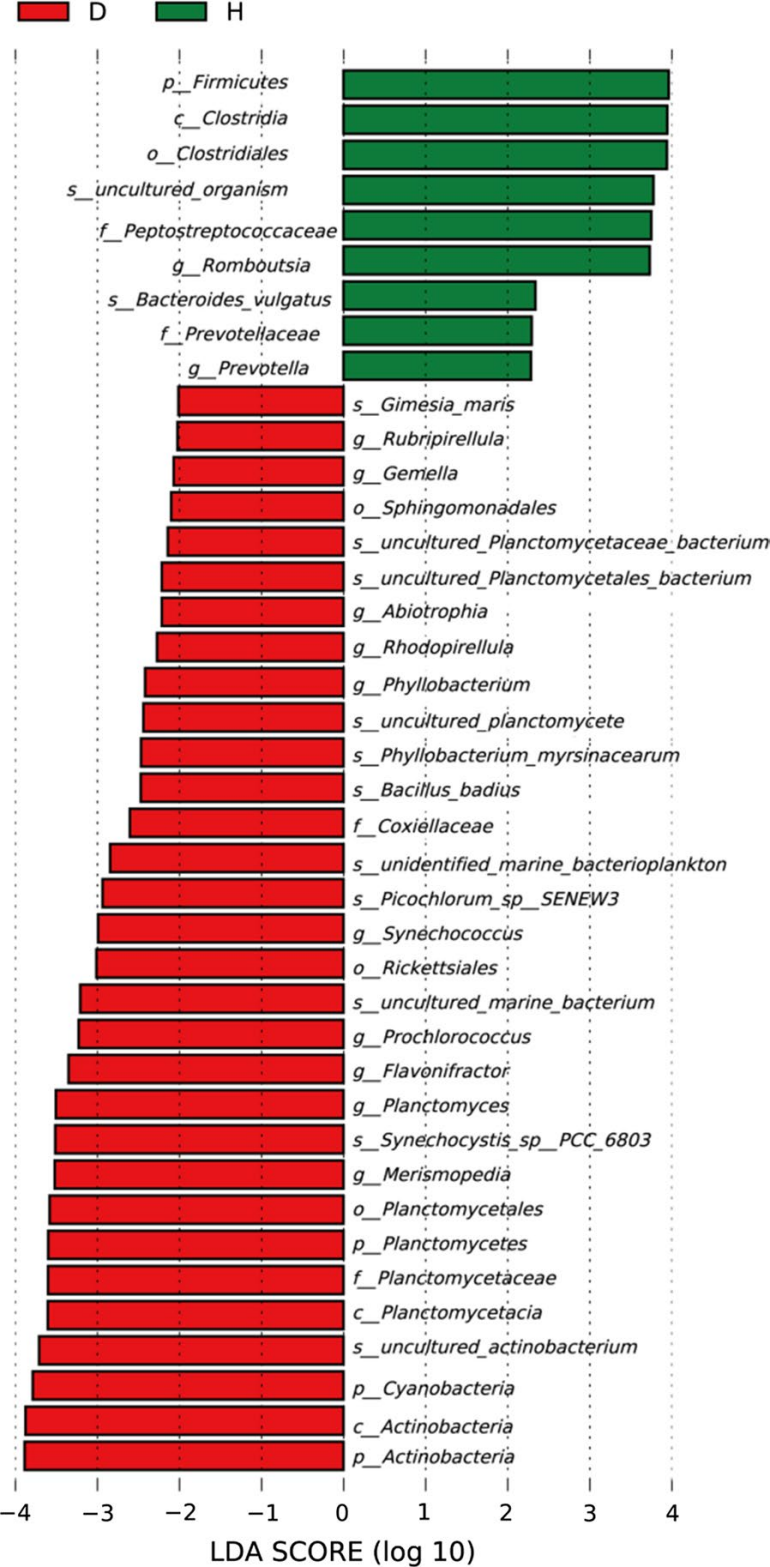
LDA scores of the intestinal microbiota in the healthy and the diseased Yunlong Grouper. A significance alpha of 0.05 and an effect size threshold of 2 were used for all biomarkers evaluated

**Fig. 4:**
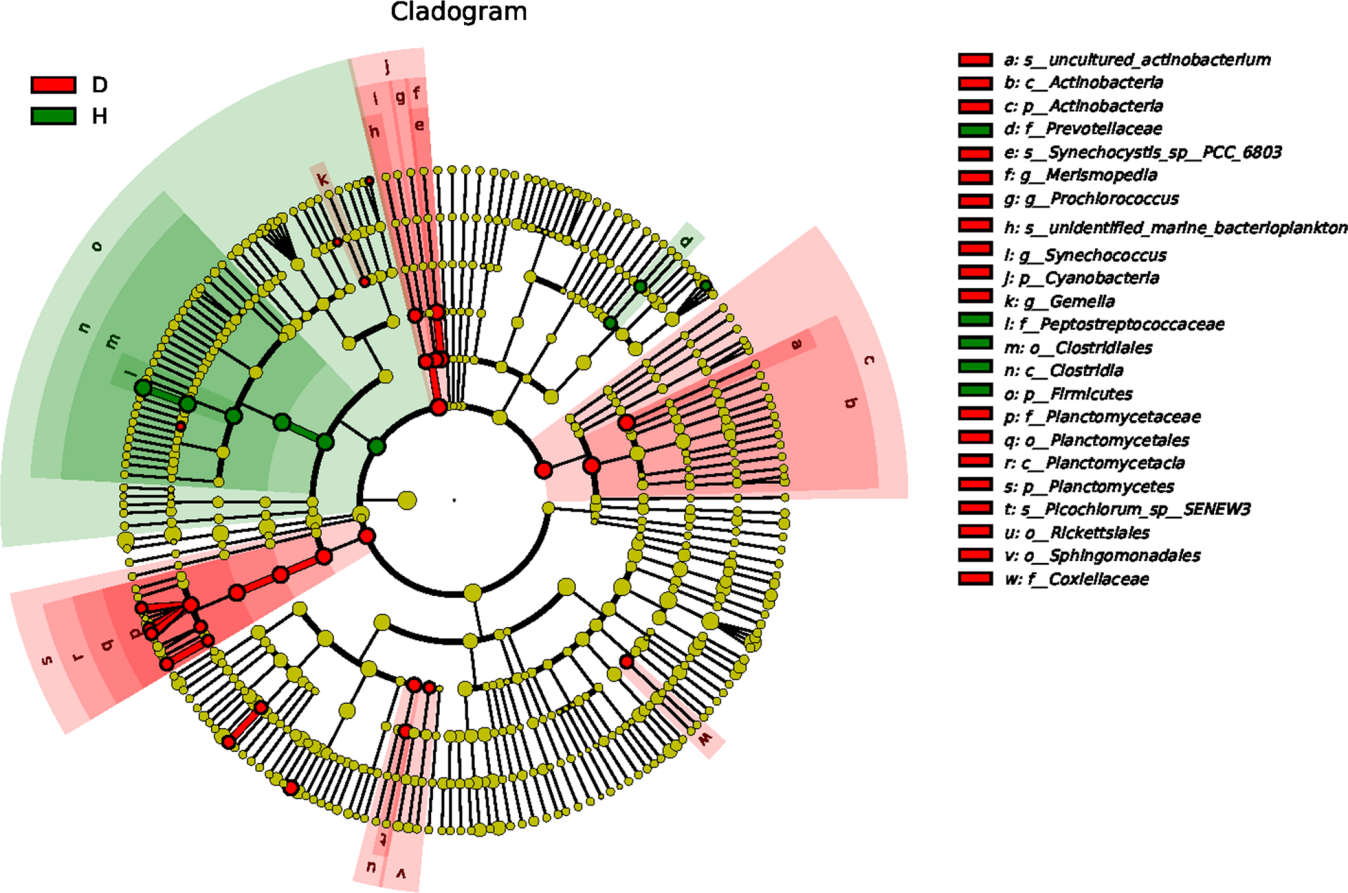
Cladogram of the intestinal microbiota via LEfSe method identifing the significantly different abundant taxa. The taxa with significantly different abundances among treatments are represented by colored dots, and, from the center outward, they represent the kingdom, phylum, class, order, family, genus, and species level, respectively. The colored shadows represent trends of the significantly differed taxa. Each colored dot has an effect size LDA score as shown in Fig. 3

LEfSe analyses (LDA values are shown in Fig. 3) were performed to determine whether there were significant differences in taxon abundance between the two fish groups (Fig. 4). For classification of phylum, the biomarkers emerging for the healthy fish group indicated *Firmicutes*; *Actinobacteria* and *Planctomycetes* were biomarkers for the diseased group; and *Actinobacteria* had the highest LDA score.

### Beta diversity analysis

**Fig. 5:**
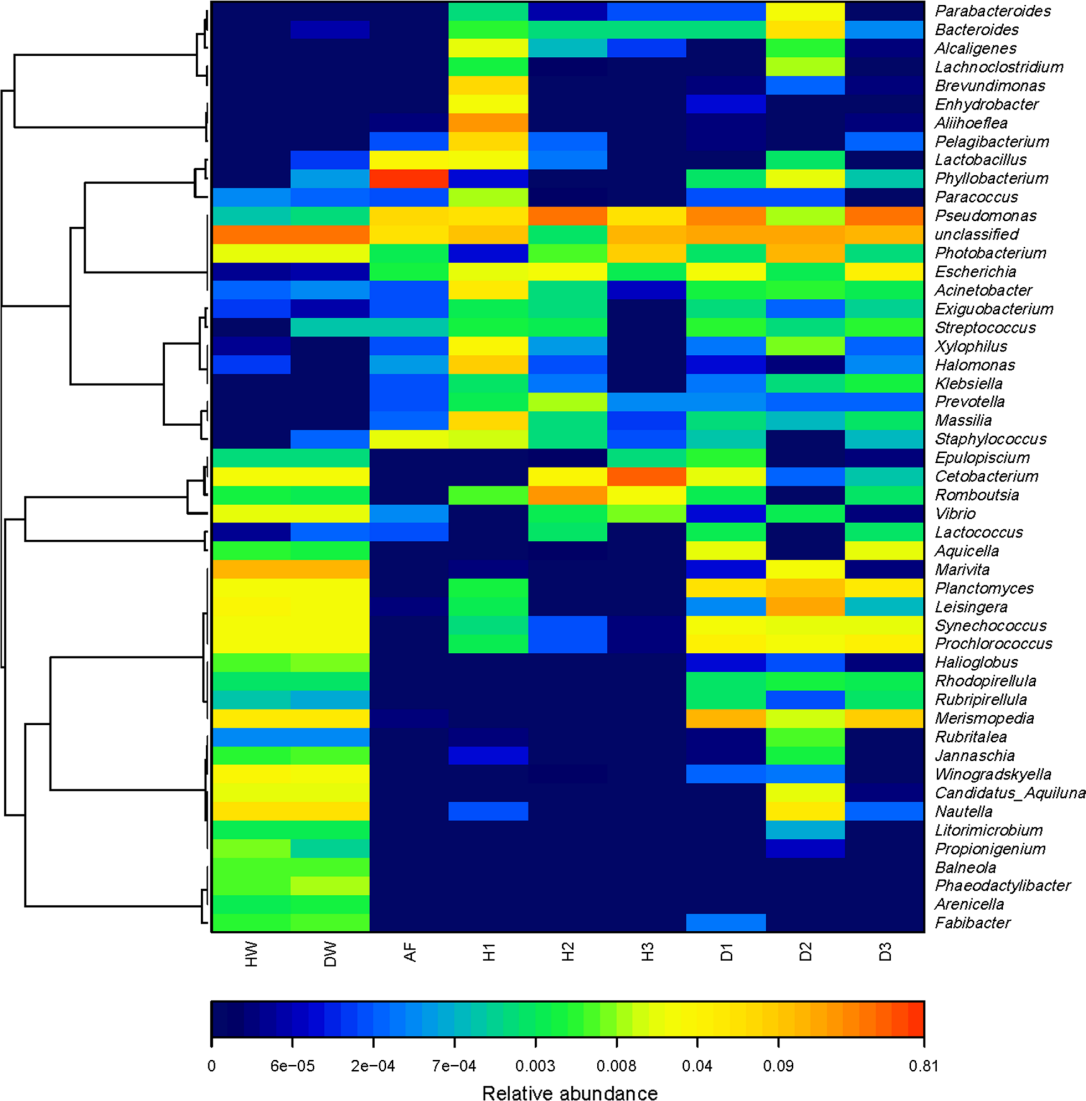
Heatmap of bacterial genera in the fish intestinal samples and the environmental samples

**Fig. 6:**
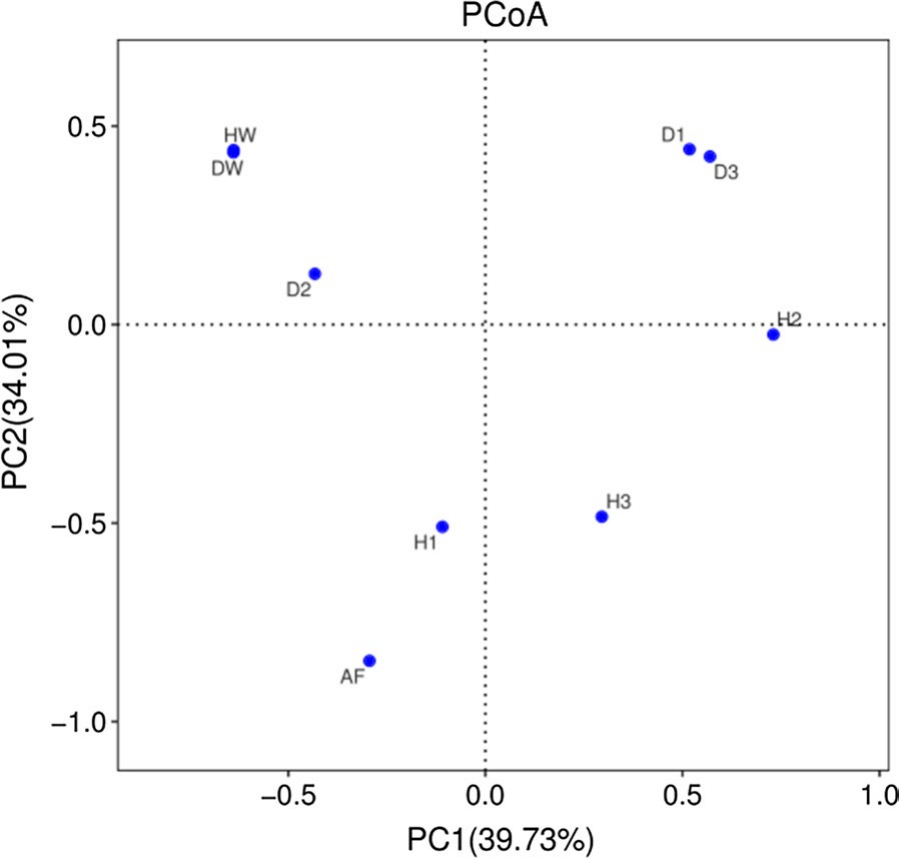
Principal coordinates analysis (PCoA) plots of beta diversity based on a weighted UniFrac distance metric

PCoA with weighted UniFrac distance and heatmap analysis were used to compare the overall composition of the microbiota between the fish intestine and the environment (Figs. 5, 6). The plot of PCoA scores shows that the environmental samples and the fish intestinal microbiota samples are separated along PC1 and accounted for 39.73% of the total variation, except for H1 and D2, which may be caused by individual differences. All the diseased fish intestinal samples and culture-water samples were above PC2. All the healthy fish intestinal tract samples and the feed sample were below PC2, and account for 34.01% of the total variation. Moreover, the composition of each intestinal microbiota sample in the healthy fish group was unique. Overall, the two PCOA axes explained more than 73% of the total variation among the different communities. The heatmap of bacterial microbiota at the genus level reflects the similarities and differences in the sample microbiota composition through color gradients and degree of similarity. The results showed that the microbiota composition was similar in the healthy and diseased fish groups.

### Functional analysis

**Fig. 7:**
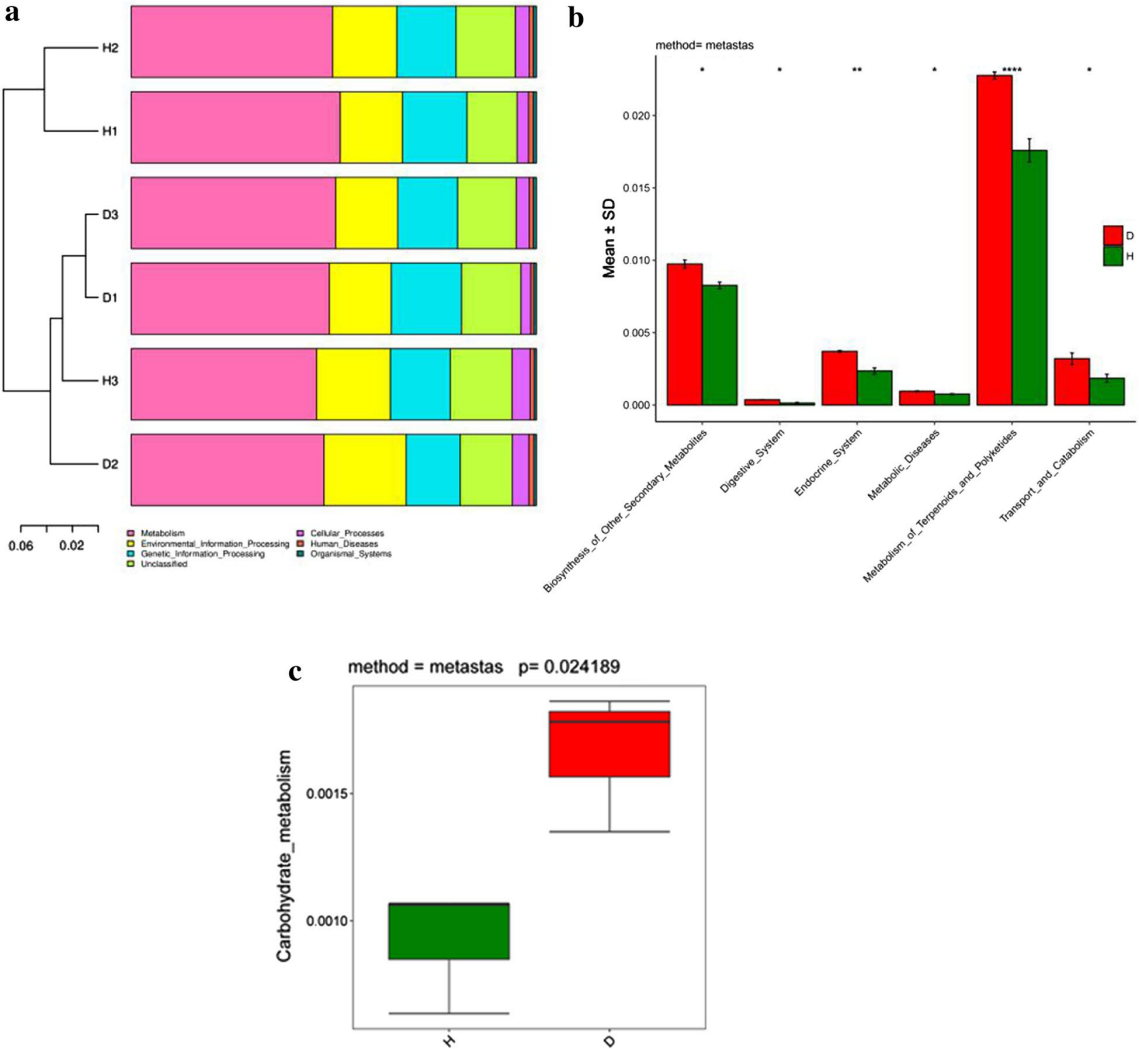
**a** The functional composition of the intestinal microbiota in Yunlong Grouper and cluster analysis. **b** In the level-2 term, significant changes are evident in intestinal microbiota KEGG pathways between healthy and diseased Yunlong Grouper, determined by the software Metastats. **c** Box-plots showed the significant predictive functions (carbohydrate metabolism) of the intestinal microbiota in both the healthy and diseased Yunlong Grouper

To analyze the functional changes of the intestinal microflora as a consequence of disease, metagenomes potential between the two groups were predicted by PICRUSt. The results revealed that high abundance of bacterial metagenome in the two groups was mainly associated with “metabolism,” “environmental information processing,” “genetic information processing,” “human diseases” and “cellular processing” in KEGG level 1 (Fig. 7a). For “metabolism,” the largest difference between the two groups was found for the level-2 terms “metabolism of Terpenoids and Polyketides” and “biosynthesis of other secondary metabolites.” Within “human diseases,” the abundance of metabolic diseases was significantly higher in the diseased group of fish than in the healthy group (Fig. 7b). In KEGG level 3, a total of 42 KEGG pathways changed significantly between the two groups by Metastats (data not shown). In the parent transport and catabolism and carbohydrate metabolism, significantly different abundances of carbohydrate metabolism were observed in the diseased group (Fig. 7c).

## Discussion

Because of its high data flux, sequencing depth and accuracy, second-generation high-throughput sequencing technology has been widely used in aquaculture, especially in the study of fish intestinal microbiota. Recent studies have found that changes in intestinal microbial diversity and imbalances of the intestinal microbiota have different effects on host fishes (Gómez and Balcázar 2010; Willing et al. 2011). Zhang et al. (2014) found that tongue sole *Cynoglossus semilaevis* with ascites displayed a significantly altered composition of the dominant intestinal microbiota. Other studies have shown that intestinal microbes can help the host to complete in regard to a variety of physiological and biochemical functions, including promoting the digestion and absorption of food, regulating the host immune system, and resisting pathogenic bacteria (Karasov et al. 2011; Forsythe and Bienenstock 2010).

To better understand the relationship between intestinal microbiota composition and fish health condition, the intestinal microbiota of Yunlong Grouper was examined. We found no significant difference between the taxonomic richness and diversity of the intestinal microbiota in diseased fish and healthy fish. The dominant phyla in the intestinal microbiota of healthy fish were *Proteobacteria* (54.02%), *Fusobacteria* (23.30%), and *Firmicutes* (20.47%); while in the intestinal microbiota of diseased fish they were *Proteobacteria* (56.21%), *Actinobacteria* (16.00%), and *Cyanobacteria* (12.61%). The composition of the intestinal microbiota of diseased fish had somewhat changed from the structure present in a healthy state. *Proteobacteria* was the most dominant phyla in the microbiota community of Yunlong Grouper. This finding is consistent with previous studies of other species of marine and freshwater fishes, including grass carp, yellow catfish, coho salmon, and rainbow trout (Wu et al. 2012, 2016a, b, c; Kim et al. 2007; Romero and Navarrete 2006). Although the occurrence of disease affected the relative abundance of *Proteobacteria* in Yunlong Grouper intestinal microbiota, it did not change its position as the most-dominant phyla in the intestinal tract. The composition of the intestinal microbiota were analyzed and compared in the healthy and diseased fish groups at the genus level; the dominant genus in the healthy group was *Cetobacterium*, with a relative abundance of 23%, while in the unhealthy group it was *Pseudomonas*, with a relative abundance of 32%, with *Cetobacterium* amounting to only 0.5%. Previous research found that *Cetobacterium*, isolated from the intestines of five different species of freshwater fish, conveyed the effects of fermented polypeptide carbohydrate and could produce vitamin B_12_, suggesting that *Cetobacterium* plays an important role in processes of digestion and nutrition in fishes (Tsuchiya et al. 2008). Furthermore, vitamin B12 can promote protein biosynthesis, acting as a growth factor in many animals, and a lack of which will generally affect growth and development (StroInsky and SchneIder 1987; Hunik 2002). Likewise, many studies have shown that vitamin B_12_ helps to maintain normal growth in fishes (Arai et al. 1972; Ikeda et al. 1988). In the current study, a comparison of the indices of body weight and body length for the two groups of Yunlong Grouper showed no significant differences between the diseased group and the healthy group. However, the growth of the diseased fish was relatively slow, with the average body weight only 54.7% that of the healthy fish, and the average body length only 80% that of the healthy fish. *Pseudomonas*, a common bacterial genus in seawater, is speciose and has great interspecies differences. The pathogenicity of *Pseudomonas* depends mainly on the physiological state of the fish body and the physical and chemical conditions of the water. Studies have shown that *Pseudomonas* infections occur in both warm- and cold-water marine fishes as well as in freshwater fishes, for example, the gill-rot disease of *Anguilla anguilla* (Fan 2001) and the Debonding disease of *Fugu obscurus* (Li et al. 2001). Thus, it can be inferred that *Cetobacterium* has the function of promoting food digestion and absorption, and general growth and development processes in Yunlong Grouper. Consequently, these bacteria can be used as probiotics in the breeding of Yunlong Grouper. However, the intestinal microbiota composition becomes disordered in diseased fish, allowing a large number of opportunistic pathogens (especially *Pseudomonas*) to proliferate; this situation may inhibit the growth of beneficial bacteria and lead to slow growth of the diseased fish. Nonetheless, the effects of *Pseudomonas* on the intestinal microbiota of fishes merits further study.

In this study, LEfSe software was used to analyze differences in the relative abundance of intestinal microbiota in different health states of Yunlong Grouper, and to estimate the effects of the differences. Bacteria from 4 phyla and 12 genera were screened. Among them, *Actinobacteria* had the highest LDA score in the intestinal microbiota of diseased fish, while in the healthy fish the score was highest for *Firmicutes*. *Actinobacteria* have the ability to biosynthesize secondary metabolites as antibiotics against invasive pathogens (Penn et al. 2009). Studies of humans have shown that patients with inflammatory bowel disease displayed a reduction in the relative abundance of *Firmicutes* and an increase of *Proteobacteria* (Blumberg and Powrie 2012; Reveco et al. 2014). That findings is consistent with our results. It is speculated that the intestinal tracts of the diseased groupers were inflamed to a certain extent, yet further study is merited on these changes in the proportions of *Actinobacteria* and *Firmicutes* in the intestinal microbiota, and their effects on the health status of fish.

Because fish live in a complex aquatic environment, so the composition of the intestinal microbiota is more susceptible to the environmental influences of the water (Ganguly and Prasad 2012). Though feed is an important factor that will affect the structure of fish intestinal microbiota (Lu et al. 2011; Dhanasiri et al. 2011; Hao et al. 2015), fish may also ingest a large number of microorganisms in the water column and sediments as well as other food items in their environment (Mandal et al. 2009). In this study, the intestinal microbiota of Yunlong Grouper was influenced by the environmental bacterial community. However, the intestinal microbiota of different individual diseased fish was relatively similar, and was highly similar to the community present in the culture water, indicating the relative susceptibility of the intestinal microbiota of diseased fish to the influence of the culture water. In contrast, every healthy fish exhibited a fairly unique composition of intestinal microbiota, which is similar to the conclusion reached by WANG (Wang et al. 2018) in a study of the intestinal microbial flora of healthy and unhealthy Atlantic salmon reared in a circular aquaculture system. In addition, we found that the feed had a greater impact on the healthy fish than on the diseased fish. Combined with the results of the intestinal microbiota and function prediction, it may be that the feeding ability of the diseased Yunlong grouper had been affected and caused them to consume less.

Previous high-throughput sequencing analysis of fish intestinal microbiota focused mainly on the microbial community structure (alpha diversity and beta diversity), composition, distribution characteristics and influencing factors, and less research has been carried out on microbial community function. More recently, PICRUSt has been widely employed for function prediction of fish intestinal microbiota (Bairagi et al. 2002; Yang et al. 2018; Lyons et al. 2017). We used PICRUSt to obtain the functional composition and determine differences in the intestinal microbiota in different health states of Yunlong Grouper. The PICRUSt analysis revealed functional richness in the intestinal microbiota of Yunlong Grouper, with many microbial functional genes related to “metabolism,” “environment information processing,” “genetic information processing,” “human diseases” and “cellular processing.” It was inferred that the intestinal microbiota of Yunlong Grouper played a role in digestion, the immune system, and adaptation to environmental changes. PICRUSt cluster analysis showed significant differences in intestinal function between the healthy and diseased fish groups. The terminal intestinal microflora of rainbow trout from different sources was found to have more sequences related to the metabolism of sugar, protein and amino acid; and it was inferred that the intestinal microflora exerts some effect on host nutritional metabolism (Lyons et al. 2017). At present, cost-efficient feeds with high carbohydrate and high fat contents are widely used in aquaculture (Xie et al. 2017). Carbohydrates need to be digested to monosaccharides prior to absorption in the small intestine (Kanehisa et al. 2017). In this study, carbohydrate metabolism were significantly greater in the intestines of diseased fish than in healthy fish. Although the abundance of genes involved in the physiological processes of carbohydrate digestion and absorption increased significantly with the occurrence of disease, the energy requirement of the immune system also increased, thus likely reducing the energy that was allocated to growth. The occurrence of disease in the Yunlong Grouper was accompanied by changes in the intestinal flora structure, which in turn results in changes in intestinal function, thereby affecting the functions related to digestion, absorption and energy metabolism. This will lead to abnormal nutrient absorption and a poor immune response in the fish, and slower growth for diseased fish as compared with healthy fish.

In this study we found no significant differences in the taxonomic richness and diversity of intestinal microbiota between healthy and diseased Yunlong Grouper, though the composition of the intestinal microbiota had certainly changed. In the intestinal microbiota of healthy fish, probiotics known to promote the digestion and absorption of food and also promote general growth and development are enriched. Pathogenic bacteria may occupy a dominant position in the intestinal microbiota of diseased fish, thus altering the function of the intestinal microbiota, in turn influencing the related functions of digestion, absorption and energy metabolism; which is more likely to lead to appearing the intestinal tract inflammation of Yunlong Grouper, and appearing the abnormal phenomena of growth of diseased fish slower than the growth of healthy fish. These results provide some basis for early detection of infectious bacterial diseases in this newly cultivated hybrid fish. Future research should increase the number of samples to eliminate individual differences, adding accuracy and reliability to the data.

## Data Availability

All sequences analyzed in this study can be accessed in the SRA database under the accession number SRP 220406 (https://www.ncbi.nlm.nih.gov/Traces/study/?acc=SRP220406). The data set supporting the conclusions of this article is included within the article. Data and materials can also be requested from the corresponding author.
